# Supplementary material for: Are carbon emissions trading and green financial instruments synergistic? -Comprehensive quantitative research based on content analysis
Source: PLoS One. 2024 Mar 7;19(3):e0298601. doi: 10.1371/journal.pone.0298601 (PMC10919601; doi:10.1371/journal.pone.0298601)
Supplement: S1 Data — (ZIP) [file pone.0298601.s001.zip › Data/地级市Z_F工作报告.zip!/┴╔─■/╔≥╤⌠╩╨/└┤╫╘╣1⁄2╓┌║┼í╛┬φ┐╦╩2╛▌═°í┐_2009.docx]

各位代表：

　　现在，我代表市人民政府向大会作工作报告，请予审议，并请市政协委员提出意见和建议。

　　一、2008年政府工作回顾

　　2008年是本届政府开局之年，也是沈阳振兴发展历程中极不平凡的一年。在省委、省政府和市委的正确领导下，市政府带领全市人民，全面贯彻党的十七大、十七届三中全会精神，深入落实科学发展观，有效应对国际国内经济形势急剧变化的严峻挑战，经受住承办奥足赛事的特殊考验，圆满完成了市十四届人大一次会议确定的各项任务，实现了经济社会又好又快发展。

　　——经济保持平稳快速增长。预计全市地区生产总值实现3855亿元，比上年增长16.3%，继续保持快速发展；全社会固定资产投资完成3000亿元，增长27%，总量在全国十五个副省级城市中位居第一；社会消费品零售总额实现1500亿元，增长22%，增幅再创历史新高；实际利用外资实现60亿美元，增长19%，总量继续保持全国十五个副省级城市第一；地方财政一般预算收入达到290.9亿元，增长26%；规模以上工业增加值实现1717亿元，增长22%，总量提前两年进入全国十五个副省级城市第一集团；规模以上工业实现利税380亿元，增长30%，经济效益明显提高。

　　——物价涨幅逐步回落。居民消费价格同比涨幅从2月份的9.2%回落到12月份的-0.8%，预计全年物价涨幅为4.4%，物价过快上涨得到有效控制。

　　——人民生活显著改善。城乡居民收入大幅度提高，预计城市居民人均可支配收入达到17290元，增长18.4%，农民人均纯收入达到8080元，增长18.7%；城镇登记失业率为3.1%，继续低于全国和全省平均水平。

　　——节能减排取得实效。预计单位地区生产总值能耗比上年下降6%，化学需氧量排放量下降6.7％，二氧化硫排放量下降3.7%，超额完成年初确定的节能减排目标。

　　——奥足赛事成功举办。圆满完成火炬传递、赛事组织、接待服务、安全保卫、城市运行等各项工作任务，为我国实现举办一届“有特色、高水平”奥运会目标作出了重要贡献，得到国际奥委会、国际足联、北京奥组委、公安部等方面的充分肯定和高度评价。

　　一年来，我们以科学发展观为指导，加快转变经济发展方式，切实保障和改善民生，着力加强生态环境建设，圆满完成奥足赛事筹办，付出极大努力，做了大量工作。

　　（一）坚持稳中求进、好中求快，努力促进经济平稳快速增长

　　面对错综复杂的国内外经济形势，我们坚持以“优化结构年”活动为主线，及时采取有针对性的政策措施，有力促进了全市经济平稳快速增长。

　　加快产业结构调整。我们以打造具有国际竞争力世界级装备制造业基地为目标，强力推进三大聚集区建设和IT、IC产业发展，促进工业整体水平全面提升，规模以上装备制造业增加值实现800亿元，增长23.2%，占全市比重达到46.6%。铁西装备制造业聚集区重点企业搬迁改造和公共平台建设取得积极进展，三一重装综采设备成套公司完成组建，重大数控机床产业园完成主体工程建设，铁西老工业基地改造成为中国改革开放三十年18个典型之一。航空经济区被批准为国家航空高技术产业基地，与中航工业集团、南航集团、加拿大庞巴迪公司的战略合作全面展开。汽车产业聚集区加快发展，通用北盛二期竣工投产，华晨宝马二期扩产项目开工建设。IT制造业产值完成270亿元，增长50%。出台《关于加快服务业发展的意见》，加大对服务业特别是现代服务业的政策扶持，服务业增加值实现1752亿元，增长15%，现代服务业比重提高到50.4%。推进大中街、太原街、近海国际物流等18个现代服务业集聚区加快发展，韩国乐天世界、香港嘉里中心、北方新天地等一批投资超百亿元重大项目聚集金廊、开工建设，金融商贸开发区被评为“中国金融生态开发区”和“最具发展潜力的区域性金融中心”。大力发展高新技术产业，规模以上高新技术产品增加值实现640亿元，增长25.3%。深入推进高档数控机床、IC装备等国家重大科技专项，R0110天然气重型燃气轮机研制成功。新增特变电工和中科仪公司2家国家工程实验室，沈阳机床、北方交通和沈阳化工研究院被确定为国家级创新型试点企业。积极推进高新技术企业进入国家代办股份转让系统。浑南高新区成为国家知识产权试点园区。高新技术产业带聚集效应凸显，高新技术产品产值占全市90%以上。软件、动漫产业快速发展，完成主营业务收入240亿元，增长50%。我市被评为“中国创新能力优秀城市”。

　　大力发展县域经济。全面落实国家强农惠农政策，出台《关于大力发展县域经济的若干意见》，投入“三农”和新农村建设资金86.8亿元，增长18.8%。粮食生产连续三年获得全面丰收，粮食总产量达到35.9亿公斤。加快发展现代农业，新增设施农业面积15万亩、高效特色农业面积45万亩，新上投资1000万元以上农产品加工项目157个，新增市级以上龙头企业43家，沈北新区被评为“中国名优食品产业基地”，农产品加工业产值实现1032亿元，增长49%。扎实推进农村市场流通体系建设，新增农民专业合作社478个。加快县域工业发展，华润雪花啤酒、康平电厂一期等一批重大项目竣工投产；新民包印、辽中铸锻等工业园区加快建设；新增法库陶瓷、苏家屯电力电器、于洪五金等4户百亿产业集群，苏家屯钢管被评为“中国县域产业集群100强”。县域地区生产总值增长23.3%，地方财政一般预算收入增长44.9%，县域成为推动全市经济发展的生力军。

　　积极推进改革开放。加快推进重点企业改革，组建市水务集团，市煤气总公司与市供暖集团联合重组，对市属大型国有企业资产实行统一监管。奥维通信等3家企业成功上市。支持非公企业做大做强，涌现出远大集团、特变电工、禾丰牧业等一批具有国际竞争力的骨干企业，非公经济已占全市经济总量的三分之二。不断深化农村改革，集体林权制度改革取得成功，农村商业银行组建工作开始启动，沈北新区村镇银行已批准开业，全市12家小额贷款公司获得批准筹办，辽中、法库信用担保中心顺利运行。扎实推进行政管理体制改革，清理62个政府议事协调机构，向四县（市）下放市级经济管理权限，建立政府绩效评估体系，市公共资源交易中心投入使用，土地交易、政府采购等全部进场交易，实行管办分离。积极稳妥实施供暖价格改革。进一步扩大对外开放，引进超千万美元项目80个，新增世界500强投资企业7家；引进投资500万元以上内资项目1820个，调入资金405亿元。全面推进地铁集团与港铁公司战略合作。近海保税物流园区建设进展顺利。出口总额实现40.5亿美元，增长22%；新签对外承包工程与劳务合作合同额11亿美元，增长2.8倍。对外交往日益活跃，成功举办东北亚发展论坛等重大国际活动。

　　推动房地产业平稳持续发展。大力实施保障性住房建设，加快工业地产、商业地产和县域房地产发展。果断出台活跃房地产市场的25条新政，鼓励住房消费、吸引开发投资、优化服务环境。实行购房补贴、降低交易税费，引导和满足居民合理扩大住房消费需求，支持居民购买自住性、改善性住房。降低首付比例，实行土地分块出让、分期付款、分割办证，减缓征收部分税费，加大金融支持力度，增强了企业投资信心，投资规模进一步扩大。全年完成房地产投资1000亿元，增长36.9%；商品房销售面积达到1320万平方米，在全国大中城市排名第二；商品房均价上涨5.2%。房地产业上缴地方税收74.7亿元，占地方税收比重达到36.6%。

　　（二）坚持以人为本、执政为民，切实保障改善民生

　　把保障和改善民生放在更加突出位置，在财政减收增支因素明显增多情况下，全市民生工程投入200.2亿元，占地方财力支出50%以上。不仅全面完成年初承诺的20件实事，而且急城乡群众之所需，增办了8件实事。

　　全面推进就业和再就业。新开发就业岗位12.3万个、公益性岗位3104个，实名制就业23.3万人。出台鼓励创业带动就业的12条政策措施，1.9万人自主创业、自谋职业。对零就业家庭、“4050”人员、残疾人等困难群体实施就业援助，18.1万困难群体人员实现稳定就业。普惠制就业培训9万人。新增农村劳动力转移输出7.6万人。落实集体合同制，积极发展和谐劳动关系。

　　着力扩大社会保障覆盖面。开展养老保险专项行动，扎实推进医疗保险参保工作，17.5万破产企业退休人员参加住院和门诊大病统筹医疗保险，城镇居民基本医疗保险参保率达到86%；新型农村合作医疗参合率达到97.7%，人均筹资标准提高到120元以上。做好被征地农民社会保障工作，6.8万被征地农民纳入养老保障体系。建立了农村低保边缘户救助制度。

　　加快保障性住房建设。确定了在全国率先解决城市低收入家庭住房困难的目标，投入23.1亿元，建成150万平方米保障性住房。为1580户农村贫困群众建房。

　　切实提高城乡居民收入水平。增加农业补贴，保证农民工工资按时足额发放，加大扶贫开发力度，多渠道增加农民收入。进一步提高企业最低工资标准。投入96.5亿元，保障和提高112万城乡低收入者等11类群体的待遇标准。落实义务教育教师津贴补贴政策，为非义务教育教师和其他事业单位人员发放临时性补贴，提高三县一市公务员津贴补贴水平。

　　努力控制物价过快上涨。设立基本生活必需品市场保障资金，加强市场监测，加大猪肉、豆油等重要商品储备，先后7次启动应急机制，保证市场供应。投入4.3亿元，实行主要副食品销售增量等补贴，对城市低保家庭、大中专院校特困生发放临时物价补贴，确保城乡低收入居民生活水平不因物价上涨而受到影响。

　　进一步改善居民生活环境。大力实施街巷路和老旧小区综合改造，投入6.5亿元，完成580条街巷路、889个住宅小区的731万平方米路面整修，小区种植树木18万株，粉刷楼体500余幢。

　　大力发展教育卫生文化体育事业。新建农村九年一贯制学校22所，超额完成三年建设百所学校任务，实现“一乡一校”，完成30所农村学校质量再提升，农村办学条件发生根本性改变。深入推进城区学校综合改革和教师交流，10个区县通过“双高普九”验收，义务教育均衡优质发展水平逐步提升。加快推进6所万人职业学校建设，改扩建70所城乡幼儿园和14所特教学校。全面免除城市义务教育阶段学生学杂费。市四院眼科大楼竣工并交付使用，奉天医院、市七院建设项目完成主体工程，沈阳中医名医馆顺利开诊。建成341家社区卫生机构，城乡医疗服务能力进一步增强。对“一宫两陵”、张氏帅府等文博场馆实施大规模修缮。全年开展群众文化活动6000余场，艺术精品不断涌现，成功举办世界奥林匹克文化展示会和国际旅游节。群众性体育活动蓬勃开展，我市体育健儿在奥运会、残奥会上取得五金两银的优异成绩。

　　（三）坚持城乡统筹、可持续发展，扎实推进生态市创建工作

　　以创建国家生态市为目标，重视资源节约和环境保护，推进城乡重大基础设施建设，切实加大节能减排工作力度，生态文明和城市环境建设迈上新台阶。城建投资完成284亿元，增长24%。

　　加强城乡基础设施建设。加快地铁建设步伐，22座车站完成主体工程，1号线开始铺轨。实施黄河大街、北陵大街等31条主次干道综合改造，五爱隧道、三好桥、沈康高速公路一期竣工通车，长白、道义等4个公交枢纽建成使用，新开、调整公交线路54条，新建农村公路500公里、客运站10个，城市交通网络体系不断完善，中心城市辐射作用进一步增强。大伙房输水配套工程开工建设，改造114处二次加压泵站及185公里给水管网，新建燃气高压外环管线100公里，城市运行保障能力进一步增强。新建农村饮水工程85处，解决了14万农村人口饮水安全问题。加快沈阳经济区基础设施建设，开工建设哈大客运专线、沈抚城际铁路，辽宁中部环线本辽段、沈阜高速公路竣工通车。

　　深入推进节能减排。坚持“上大压小”，开工建设国家重点节能工程金山热电项目。推广地源热泵、煤层气等清洁能源，新增地源热泵应用面积1618万平方米。淘汰一批落后生产能力，搬迁重点污染源企业31户，关停高污染落后产能企业150家，完成沈海热电等5家企业脱硫工程。开展再生能源利用，沈阳再生资源产业园被列入第二批国家级循环经济试点单位，老虎冲垃圾场沼气发电工程并网发电。

　　加快生态市创建工作。开工建设日处理能力60万吨南部污水处理厂，建成11个区县（市）污水处理厂和31个村镇污水处理设施，新民、康平垃圾填埋场建成并投入使用。沈北30万吨铬渣无害化处理工程投入运行。设立33个生态保护地，受保护地区覆盖率达到30.9%。启动“环城水系改造工程”，实施浑河沈阳城市段改造。完成19条林阴大道建设，建成区绿地率提高到38.3%。26个乡镇通过国家环境优美乡镇验收，东陵区在东北地区率先通过国家生态区技术核查，铁西区荣获“2008联合国全球宜居城区示范奖”，沈阳成为“国家园林城市”。

　　（四）坚持科学组织、周密安排，全力筹办奥足赛事

　　奥足赛是沈阳有史以来承办规格最高、规模最大的国际体育赛事，全市上下齐心协力、连续奋战，圆满完成了奥足赛事筹办和组织工作，充分展示了沈阳综合实力和城市文明形象。

　　精心组织奥足赛事。用最短的时间，高质量建设了设施一流、功能完善的比赛场馆，奥体中心体育场建设创造国际、国内7项第一。组成12.1万志愿者队伍，精心编制工作方案和应急预案，多次进行实战演练，实现了场馆运行与城市运行、外围保障的无缝衔接。奥足赛期间，共接待外宾1082人、148家中外媒体记者1337人次，赢得了各参赛队、奥林匹克大家庭成员和国内外媒体的一致好评。

　　全力保障城市运行。新增、更新1700辆环保公交车，开通40条午夜公交专线和12条奥运公交专线，组织5000辆具备叫车功能的出租车，满足了赛事期间的运力要求。对涉奥场馆、接待宾馆、重点街路、城市出口、铁路沿线和宗教场所进行环境综合整治，实现城市环境美化、亮化、净化。加强供水、供电、供气、通信、排水、防汛等各项公共服务，为赛事成功举办提供了有力保障。

　　切实维护社会稳定。加大信访稳定工作力度，依法处置“蚁力神问题”，市财政筹资30.7亿元，盘活土地等资产，全面解决了“荷兰村”占地补偿、浑河大市场业户清退和“天山福园”等历史遗留问题，维护了群众切身利益。坚持把奥运安保工作作为头等大事来抓，建立了警民联手、军地协作、专群结合的工作体系，调集3.7万安保力量，确保了奥运火炬传递及奥体中心、奥运村、媒体村等重点部位和国内外政要等重点人员的绝对安全。全力以赴、严查严管，保证了食品药品安全，有效防控了各类重大传染病的发生和流行。全面加大涉恐专案侦查力度，通过“大巡逻、大防控”，防止重点人员、不安全车辆和危险物品进入沈阳、流入北京。奥运期间，我市“大事没出，小事也没有出”，实现了平安奥运目标。

　　四川汶川特大地震发生后，我市立即组织救援队伍赶赴灾区抢险救援，迅速购置大批救灾物资发往灾区，累计为灾区捐款2.4亿元。积极收治伤员、接收学生，按期完成1万套过渡安置房建设任务，对口支援灾区恢复重建，体现了中华民族守望相助的传统美德。

　　此外，人口与计划生育、统计、监察、审计、物价、参事、民族、宗教、侨务、广播、电视、新闻、出版、文化执法、工商行政管理、质量技术监督、食品药品监督、气象、民兵、预备役、双拥、防震减灾、人防、档案、修志、文史、红十字会、残疾人事业和老龄工作也都取得了新成绩。

　　各位代表，过去一年，在国际经济形势急转直下、国内经济运行困难明显增多的错综复杂情况下，我们能够取得经济社会又好又快发展的成绩，确实来之不易，应该倍加珍惜。这是省委、省政府和市委正确领导的结果，是市人大、市政协监督和支持的结果，是全市人民拼搏奋斗的结果。我代表市政府，向全市各族人民致以亲切问候和崇高敬意！向人大代表、政协委员，各民主党派、工商联、各人民团体和各界人士，向参与沈阳发展建设的中央和省在沈单位、驻沈部队指战员、武警官兵、港澳台同胞、海外侨胞及国际友人表示衷心感谢！

　　在充分肯定成绩的同时，我们也必须清醒地看到，受国际金融危机持续蔓延和世界经济增长明显减速的影响，目前我市经济运行中的困难急剧增加，社会发展和政府工作中还存在不少问题。一是经济下行压力加大。地区生产总值、工业增加值、利用外资等主要经济指标增速大幅回落，全社会用电量、铁路货运量增幅明显下降。二是部分企业经营困难。市场环境趋紧，企业订单减少、销售不畅、利润缩减状况开始显现。三是就业形势严峻。部分企业出现待岗现象，预计今年全市需安排就业人员将明显增多，就业再就业压力很大。四是政府自身建设和管理需要加强。政府职能转变还不到位，社会管理和公共服务比较薄弱，一些部门职责交叉、办事效率低下。对此，我们必须高度重视，切实加以解决。

　　二、2009年政府工作安排

　　今年是新世纪以来我市经济发展面临困难最大的一年，也是蕴含重大发展机遇的一年，做好政府工作意义十分重大。尽管当前受国际金融危机影响，经济发展存在许多不确定因素，但我市仍处于振兴发展的重要战略机遇期。我们要科学判断形势，化危机为机遇，变挑战为动力，以百倍的信心和扎实的工作，推动科学发展，促进社会和谐。

　　今年政府工作的总体要求是：认真贯彻党的十七大、十七届三中全会和中央经济工作会议精神，深入落实科学发展观，紧紧围绕完成“五大任务”、实现“三大目标”，立足扩大内需保持经济平稳较快增长，抓好重大项目建设，加快转变发展方式，继续优化经济结构，大力推进改革开放，加强生态环境建设，着力保障改善民生，促进经济社会又好又快发展。

　　全市经济社会发展主要预期目标是：地区生产总值增长14%，地方财政一般预算收入增长16%，全社会固定资产投资增长15%，社会消费品零售总额增长15.5%，实际利用外资增长10%，城市居民人均可支配收入和农民人均纯收入分别增长14%，居民消费价格涨幅控制在4%以内，城镇登记失业率控制在4.2%以内，单位地区生产总值能耗下降4.5%，主要污染物排放总量减少3%。

　　（一）积极扩大内需，促进经济平稳较快增长

　　准确把握积极财政政策和适度宽松货币政策的导向，紧紧抓住国家和省扩大内需促进经济增长的难得机遇，进一步加大投资力度，优化投资结构，着力扩大消费需求特别是居民消费需求，实现投资消费共同拉动经济平稳较快增长。

　　加大固定资产投资力度。通过增加政府投资带动社会投资，鼓励引导民间投资，全社会固定资产投资实现3450亿元。加快铁路、公路、机场、地铁、城市道路等重大基础设施建设；加快重大水利工程、设施农业等农业和农村基础设施建设；加快先进装备制造业、现代服务业和高新技术产业重大项目建设；加快节能减排、污染治理和生态建设；加快城乡保障性住房、医疗卫生、教育、文化等重大民生工程和社会事业建设。

　　积极扩大消费需求。把扩大消费与完善收入分配政策、发展服务业、扩大就业结合起来，积极鼓励居民消费，社会消费品零售总额达到1733亿元。千方百计增加城乡居民收入，特别是提高中低收入群体收入，不断提高消费能力。改善居民消费预期，增强消费意愿。大力培育消费热点，鼓励住房、汽车等大宗消费，发展社区商业、物业、家政等服务性消费，开发旅游、文化、体育健身等热点消费。积极开拓农村消费市场。完善相关消费政策，优化消费环境，加强城乡消费设施和服务体系建设。促进商贸服务业提档升级，增强区域集聚和辐射能力。

　　扩大内需，要坚持保持增长速度和提高质量效益相统一，推进结构升级和扶持就业创业相协调，保障改善民生和促进经济发展相适应，加强政府调控和发挥市场机制作用相促进，拉动当前经济增长和增强经济长期发展后劲相结合，努力实现好中求快、又好又快发展。

　　（二）突出抓好“三农”工作，开创农村改革发展新局面

　　全面贯彻落实十七届三中全会精神，把解决好“三农”问题作为全部工作重中之重，巩固和加强农业基础地位，以制度建设和机制创新推动农村改革发展，促进农民持续增收，加快形成城乡经济社会发展一体化新格局。

　　继续深化农村改革。在保持农村家庭承包经营制度长久不变的前提下，积极推行多种方式土地流转，在具备条件乡镇建立土地流转中心，发展多种形式适度规模经营。培育农民新型合作组织，新发展农民专业合作社200个。改善和创新农村金融服务，加快农村商业银行、村镇银行等金融机构组建步伐，设立一批小额贷款公司，健全县域担保体系，开展政策性农业保险试点。增强乡镇政府社会管理和公共服务职能，创新农业经济区管理体制和运行机制。继续深化集体林权制度改革。

　　提高农村经济综合实力。重视粮食生产和农产品有效供给，优化品种结构，提高单产水平，稳定粮食总产。大力发展现代农业，扩大设施农业和高效特色农业规模，推进规模化标准化健康养殖，推动农业经济区主导产业升级。强化农业生产、流通、科技和信息服务，加快农业标准化建设，建立健全动植物防疫体系和农产品质量安全检验检测体系，形成10个农业品牌生产基地。扶持壮大龙头企业，新上投资1000万元以上农产品加工项目100个。加快东陵浑河南岸产业园、新民经济开发区等工业园区建设，康平塑编产业集群实现百亿产出。深入推进“万村千乡”市场工程和“新网工程”，建设区域性中心市场。

　　加强农业和农村基础设施建设。加快以农田水利为重点的农业基础设施建设。积极推进14个市级以上中心镇建设，新建和改建2个大型农村枢纽客运站，新建1000个农村候车廊，建设500公里农村公路。加快电网、通信等基础设施建设。发展户用沼气9000户，建成3个垃圾处理场、10个垃圾转运站和10个村镇污水处理设施。投入6.3亿元，基本解决90万农村人口饮水安全问题，让广大农民喝上放心水。

　　加快发展农村公共事业。积极化解农村“普九”债务，完成74所九年一贯制学校质量再提升工程，改造65所乡镇中心幼儿园，推进2所职教中心建设。新建60个乡镇文化中心，改善107所乡镇卫生院医疗条件，新建和改扩建22所农村区域性中心敬老院。

　　（三）大力发展先进装备制造业，加快工业结构优化升级

　　制定出台振兴装备制造业的政策措施，加快三大聚集区建设，以重大项目带动产业优化升级，提高企业素质和市场竞争力，促进工业由大变强，增强工业对经济增长的拉动作用。工业固定资产投资增长15%以上，规模以上工业增加值增长18%。

　　着力提升铁西装备制造业聚集区建设水平。积极争取将聚集区发展列入国家“十二五”规划。加快高档数控机床、通用装备、重矿机械、输变电装备等重点产业发展，推进地铁装备产业化，扶持核电、风电等新兴产业。支持、推动装备制造骨干企业拓宽产品领域，调整产品结构，提高核心竞争力和盈利水平。加大公共制造、检测、物流等服务平台建设力度。加快安川电机、输变电产业园等重大项目建设。启动沈阳大工业区起步区建设，积极发展装备制造配套产业。

　　加快推动航高基地发展。把航空制造业作为发展先进装备制造业的重点，加大政策扶持力度，突出抓好50平方公里核心区基础设施建设，深入推进与中航工业集团、南航集团、加拿大庞巴迪公司的战略合作，加快C系列飞机、Q400飞机大部件转包、ARJ-21发动机总装、南航维修基地、空港物流等重大项目建设。

　　大力推进汽车产业聚集区建设。加大欧盟经济区开发建设力度，加快推动通用北盛二期达产达效，支持华晨宝马二期扩产改造、华晨金杯A级车扩产项目建设。积极推出新车型，创建整车自主精品品牌，加强零部件配套，切实扭转汽车生产下滑局面，努力打造产出双千亿元的汽车整车和零部件产业集群。

　　不断壮大IT、IC产业。以晨讯、德信、新邮通等企业为重点，加快通信产业发展。支持沈阳芯源、沈阳新松机器人等IC装备企业做大做强。重点推进奥园动漫产业园、沈阳国际软件园等产业基地建设，积极引进IBM、印度软件联盟等研发中心，加快发展软件产业。

　　切实增强自主创新能力。加快浑南国家级高新区建设。推进科技保险试点工作。深入实施IC装备、R0110合成气重型燃气轮机等重大科技专项。加强产学研合作，强化与大专院校、科研院所对接，支持企业技术中心、工程中心、重点实验室建设，引进40个具有国际竞争力的技术和管理团队。推进专用数控机床、生物制药等高新技术产业化，培育一批拥有自主知识产权的高技术产业群。重点建设沈阳超级计算中心等公共研发平台。出台鼓励购买和使用首台首套重大技术装备优惠政策，发展重大成套设备。支持重点骨干企业通过国际并购、收购发明专利，提高国际竞争力。

　　（四）优先发展现代服务业，提升服务业整体水平

　　把服务业发展放在更加突出位置，大力发展生产性服务业和生活性服务业，规范提升传统服务业，积极打造东北区域金融、物流、现代商贸、休闲旅游中心，进一步提高中心城市综合服务功能。现代服务业占服务业比重提高到52%以上。

　　促进房地产市场稳定健康发展。落实好25条房地产新政，做好房地产市场运行分析，健全预警预报机制。引导和调控房地产市场走势，稳定市场信心和消费预期。改善住房供应结构，规范市场交易，加快发展二手房市场和住房租赁市场，精心办好房交会。房地产投资实现1150亿元，切实发挥房地产业在扩大内需促进增长中的引擎作用。

　　大力发展金融业。依托沈阳金融商贸开发区、沈阳金融街，加快银行、保险、证券、期货和信托投资等国内外金融机构聚集，优化金融发展环境，全面提升区域金融服务水平。

　　积极发展现代物流业。加快建设沈阳近海东北亚国际物流中心，重点推进营口港干港建设，保税物流中心封关运行。积极推进东北农副产品交易中心、国药物流等重点项目建设。

　　全面提升商贸服务业。大力发展连锁经营、电子商务等现代流通方式，进一步优化市场结构和网点布局。高水平建设西塔街、三好街、北二路、沈阳路等一批特色街区。推进五爱市场、南塔市场、塔湾农产品市场等一批商品交易市场转型升级。

　　加快发展文化旅游业。发挥棋盘山国家级文化产业示范园区带动效应，开工建设华强文化科技产业基地、中国卡通娱乐城，精心打造“一宫两陵”、张氏帅府、世博园、浑河沿岸风景区、陨石山风景区等城市品牌，推出反映关东文化特色的大型实景演出。加快建设沈阳经济区旅游集散中心，把我市建设成为面向东北亚、最具吸引力的旅游目的地城市。

　　推进服务业集聚区建设。进一步完善金廊银带和大中街、大太原街现代商贸区等重点集聚区规划，加快龙之梦亚太中心、华府天地二期、沈阳国际金融中心等超百亿元重大项目和140个投资10亿元以上项目建设，着力引进一批世界知名企业，将金廊建设成为我市现代服务业的集中展示区。

　　（五）深化改革开放，完善有利于科学发展的体制机制

　　加快重点领域和关键环节改革，进一步扩大对外开放，努力破解制约经济社会发展重点难点问题，为科学发展不断注入新鲜活力和持久动力。

　　大力推进企业兼并重组。抓住当前企业优胜劣汰、兼并重组的有利时机，鼓励行业龙头企业、优势企业兼并重组落后企业、困难企业；鼓励优势企业强强联合，提高规模效益；鼓励关联企业、上下游企业联合重组，实现一体化经营。深化沈鼓、北方重工等重点国有企业股份制改革，支持沈阳机床、远大等骨干企业跨地区、跨行业、跨所有制战略重组，打造世界一流跨国集团。

　　鼓励支持非公有制经济发展。适时召开全市非公有制经济大会，出台政策措施，加强对中小企业的财税支持，放宽市场准入，支持民间资本参与国有企业改革。完善中小企业社会化服务体系，健全担保体系及担保风险补偿机制，扩大直接融资渠道，努力解决融资难问题。支持中小企业转型升级，引导中小企业为装备制造重点企业开展配套服务，提高市场竞争力。

　　深入实施财政金融体制改革。深化财政预算制度改革，强化国有资本经营预算管理，削减行政事业性收费，进一步完善市、区县（市）财政体制。加强财政税收管理，依法加强税收征管，规范非税收入，做到应收尽收。优化财政支出结构，严格控制一般性支出，对公务购车和用车经费、会议经费、公务接待费、出国（境）经费等支出实行零增长，严格控制以各种名义兴建楼堂馆所；继续增加教育、文化、就业、医疗卫生、社会保障等民生支出，加强对“三农”、非公经济、节能减排、生态环境保护的支持；市财政从今年起至2012年，每年安排10亿元产业专项资金，加大对重点产业、企业和重大项目的扶持力度。深化投融资体制改革，加快组建城建投融资平台，探索发行企业债券，推进盛京银行等企业上市，支持沈阳联合产权交易所做大做强，积极争取国家装备制造产业投资基金落户沈阳。

　　积极推进水价和环保收费改革。适时调整供水价格，推行非居民用水超定额累进加价制度，实施污水、垃圾处理和排污收费改革。

　　拓展对外开放广度和深度。提高利用外资水平，引进超千万美元重大外资项目50个以上；引进投资500万元以上内资项目1800个，调入资金460亿元。深入推进地铁集团与港铁公司合作。保持对外贸易稳定增长，支持高新技术产品、机电产品、农产品出口，大力发展服务贸易，加快推进服务外包基地建设。鼓励有条件的企业“走出去”合资合作、承揽大型工程，加强劳务输出。精心打造东北亚发展论坛、制博会等对外开放平台，办好沈阳金融服务外包交易会。

　　加快推进沈阳经济区一体化进程，加强重大基础设施建设，优化生产要素配置，形成综合承载能力强、辐射作用大的城市群，提高整体对外开放水平。

　　（六）加快城市建设步伐，提高生态环境质量

　　全面加强节能、节水、节地、节材和资源综合利用，大力推进城乡基础设施和生态环境建设，加快建设宜居生态城市。全年城建投资360亿元。

　　建设一批基础设施重大项目。积极推进哈大、沈丹、沈铁、西部出海铁路和沈阳新客站建设。重点推进沈康高速二期、沈彰高速、沈抚2号公路、西北连接线、沈本开发大道建设。改造沈阳桃仙国际机场1号航站楼，新建3号航站楼。加快推进地铁1、2号线一期工程，确保1号线全线开通，积极争取国家批准3、4、5号线开工建设。努力消除城市道路交通瓶颈，实施45处城市桥梁和63项市政道路改造工程。开工建设沈阳国际展览中心。做好金廊银带、二三环间等重点地区基础设施配套。完成大伙房水库输水配套工程建设。

　　加强生态环境建设。优化长白岛生态环境，努力建设现代化生态岛城。大力推进浑河生态提升工程，全面建成治理辽河流域的25个污水处理厂，实施卧龙湖“引辽济湖”工程。开展绿色村庄及沈西北边界防风阻沙带建设，全市农村完成绿化和植树造林40万亩。力争创建1个国家生态区县和20个国家环境优美乡镇。

　　加大节能减排工作力度。突出抓好工业、交通、建筑三大领域节能工作，加快重点节能工程、重大减排项目、重点污染治理工程建设。实施87个节能降耗项目，支持东药、红梅、沈化等高排放企业整体搬迁改造。积极推进IGCC多联产、中弘再生资源等节能示范项目建设。完成沈海热电等7家热电企业和46家单位110台燃煤锅炉脱硫工程。严格执行新建项目环保审批制度，实施重点污染源在线监测。

　　（七）着力保障改善民生，全面加强社会建设

　　进一步加大民生工程投入，积极推进基本公共服务均等化，继续为城乡群众办好20件实事，努力办成一批人民群众热切期盼的大事实事，把扩大投资的民生工程办成人民群众真正得到实惠的民心工程。

　　努力增加城乡居民收入。落实粮食最低收购价格、农资综合直补、良种补贴、农机具购置补贴政策，确保农民工工资按时足额发放，做好25个重点贫困乡镇扶贫开发工作，促进农民价格增收和务工增收。建立企业职工工资正常增长和支付机制，完善和落实公务员津贴补贴政策，推进事业单位工资改革。投入117亿元，全面保障和提高企业退休人员、城乡低保户、低保边缘户等11类低收入居民及部分特殊群体共计122.8万人的待遇标准。

　　千方百计扩大就业。今年是就业形势非常严峻的一年，要实施更加积极的就业政策，调动企业、劳动者和社会各方面力量，全力保持就业形势稳定。发挥政府投资和重大项目带动就业作用，积极发展劳动密集型产业和中小企业，扩大失业保险基金使用范围，完成实名制就业25万人。全面推动以创业带动就业，鼓励自主创业、自谋职业，切实帮助创业者解决场地、资金等问题。做好公共就业服务工作，扩大普惠制就业培训范围，全年培训10万人。重点做好高校毕业生、复员转业军人等重点人群就业工作，加大就业援助力度，开发公益性岗位，做好零就业家庭等困难群体就业帮扶工作。帮助企业稳定就业岗位，鼓励企业开展待岗职工培训，防止大规模集中裁员。在39个中心乡镇建立就业和劳动保障公共服务平台，加强对农民工流动就业的引导和服务，对返乡创业的农民工给予政策扶持。

　　完善社会保障体系。扩大养老、医疗、失业、工伤、生育保险覆盖面，改进和完善事业单位养老保险办法，探索建立农村养老保险制度，做好大学生参加城镇居民基本医疗保险工作，新型农村合作医疗筹资标准提高到150元以上。进一步健全城乡社会救助体系，提高救助标准。积极发展社会福利、优抚安置、慈善和残疾人事业，建立和完善城乡养老服务体系。加大住房保障力度，投入29.2亿元，对5.6万户城市低收入家庭购买经济适用房实施补贴，为4500户农村贫困家庭建房，全面完成城市保障性住房建设任务，在全国率先解决城乡低收入家庭住房困难。

　　加快发展教育卫生文化体育事业。全面完成城区学校综合改革，启动“班班通”工程，完成10所普通高中标准化建设，建成4个职业教育公共培训基地，改造100所惠民幼儿园，提升特教学校办学水平，支持高校加快改革发展，不断完善终身教育体系。做好重大疾病防治工作，提高公共卫生事件应急处置能力，完善社区卫生服务功能，新建和改扩建15个社区卫生服务中心，启动城乡药品配送体系建设，全面完成6所市属医院建设和市儿童医院改扩建一期工程。加快公益性文化事业和经营性文化产业发展，新建艺术中心、少儿图书馆、博物馆、规划展示馆等一批公益性文化设施。加强公共体育设施建设，广泛开展全民健身活动，办好第十二届沈阳市全运会。

　　全面提高食品药品质量和安全生产水平。深入开展食品药品安全专项整治，加强对食品药品生产流通全过程监管，加大重点产品抽查力度，引导企业增强社会责任意识，坚决杜绝不合格产品进入市场。进一步加强安全生产治理整顿，全面排查安全隐患，从管理、投入、制度建设等方面多管齐下，严肃查处各类事故，有效防范和坚决遏制重特大安全事故发生，确保人民群众生命财产安全。

　　切实维护社会和谐稳定。推进社区服务网络建设，努力构建和谐社区。正确处理人民内部矛盾，进一步健全社会矛盾纠纷调处机制，下大气力解决群众反映强烈的信访突出问题，有效防范非法集资、征地拆迁等可能引发的群体性事件。加强社会治安综合治理，不断完善社会治安防控体系，依法打击各类犯罪行为，增强人民群众安全感。

　　深入开展国防教育和双拥宣传教育，做好双拥共建和优抚安置工作，努力争取全国双拥模范城七连冠。

　　（八）努力转变政府职能，推进服务型政府建设

　　贯彻落实国家扩大内需方针，保持经济平稳较快增长，是对政府驾驭复杂局面能力和执政能力的重大考验。要坚持依法行政，强化公共管理和社会服务，进一步提高政府效能。

　　加快行政管理体制改革。适时启动政府机构改革，理顺部门职责关系，完善绩效评估考核机制。深化行政审批制度改革，进一步取消和调整审批项目，加强对已取消审批项目的后续监管。继续深化公共资源交易平台建设。

　　努力提高政府决策水平。深入实际调查研究，坚持问政于民、问需于民、问计于民，加强决策的科学化、民主化、法制化，使我们提出的发展思路、作出的工作部署、出台的政策措施更加符合实际、更加符合人民群众愿望。

　　积极为企业排忧解难。制定并出台帮助企业应对国际金融危机的政策措施，从财税、金融、产业等方面支持企业发展。清理和规范各种收费，减轻企业负担。加强和改进服务，帮助企业渡过难关。

　　切实转变工作作风。各级领导干部要牢固树立正确政绩观，多干打基础、利长远的事，把精力用在谋发展上，把心思放在办实事上，敢于承担责任，敢于接触矛盾，敢于动手解决问题，埋头苦干，狠抓各项工作落实。

　　大力加强廉政建设。把反腐倡廉建设放在更加突出位置，旗帜鲜明地反对腐败。扎实推进惩治和预防腐败体系建设，强化权力运行制约和监督，防止违法违纪案件和侵害群众利益问题发生。

　　各位代表，逆水行舟，不进则退。应对国际金融危机，保持沈阳经济平稳较快增长，任务繁重而艰巨，让我们在省委、省政府和市委的领导下，锐意进取，顽强拼搏，为实现沈阳老工业基地全面振兴而努力奋斗！
